# Use of cell phone data to correct Île-de-France population estimates and SARS-CoV-2 incidence, July to September, 2021: a proof-of-concept exercise

**DOI:** 10.2807/1560-7917.ES.2025.30.22.2400530

**Published:** 2025-06-05

**Authors:** Arnaud Tarantola, Mohamed Hamidouche

**Affiliations:** 1 Direction des Régions, Santé Publique France, Saint-Maurice, France

**Keywords:** mobile phone, cell phone, population, incidence, rates, epidemic, epidemiology, Covid-19, SARS-CoV-2

## Abstract

**Background:**

During the COVID-19 pandemic, Santé publique France (SpF) published incidence (SpF*i*) rates based on census denominators. Denominators using cell phone connection (CPC) data can better reflect the population present and seasonal mobilities.

**Aim:**

Given uncertainties regarding the actual number of Île-de-France (IdF) residents present in IdF during summer 2021, we aimed to better approximate true incidence rates from positive SARS-CoV-2 tests in IdF using CPC-derived population denominators.

**Method:**

This longitudinal study used the daily number of positive tests (PCR and Ag) on IdF residents in IdF as the numerator and the estimated resident population present in IdF at midnight as the denominator. We computed the mean corrected incidence rate (MCIR) per moving week between 4 July and 9 September 2021.

**Results:**

The MCIR showed higher incidence rates than initially estimated, especially during August when residents had left IdF for the holidays. Incidence rates reached a peak on 16 August when the SpF*i* rate per moving week was 200.9 per 100,000 compared with 315.6 per 100,000 with the MCIR, representing a 57% increase.

**Conclusion:**

Using local SARS-CoV-2 testing data and real-time population denominators, we showed that indicators using non-geographically referenced test results and fixed population denominators that ignore seasonal mobility can significantly underestimate incidence rates in IdF. New data sources using CPC data provide the opportunity to calculate more accurate and dynamic incidence rates and to map epidemics more precisely and in real time.

**Key public health message**

**What did you want to address in this study and why?**

During COVID-19, high population mobility over short periods such as holidays led to false estimates of case numbers. Instead of census data, we used cell phone data to better estimate the population actually present in Île-de-France (IdF) and computed a more accurate estimation of SARS-CoV-2 incidence in IdF during the period 28 June–9 September 2021.

**What have we learnt from this study?**

The corrected rates based on cell phone data showed a more intense epidemic than initially estimated, especially during the month of August. Using local testing data and real-time population denominators, we showed that indicators computed using fixed population denominators ignored seasonal mobilities and significantly underestimated incidence rates in IdF by up to 57%.

**What are the implications of your findings for public health?**

This approach offers better estimations and fine-scale mapping of the epidemiology of infectious diseases during periods of high population mobility or mass events.

## Introduction

Epidemics and outbreaks are defined by comparing case counts to an expected number of cases. However, case counts and other numerators only provide a simple understanding of the epidemiological context. To compare case counts across time, space and settings, epidemiologists use denominators to compute proportions, ratios or incidence rates. Since Graunt and Petty’s documentation of deaths, causes of death and births as numerators in the 17th century [1], epidemiologists have endeavoured to document accurate population denominators [2]. In most settings, census data meet the denominator requirements of epidemiologists and public health practitioners, especially if the population at risk is large and does not vary too greatly or too quickly over time. In some settings, however, census data are unreliable for accurate computation of incidence rates because of unavailable population estimates or rapid population movements [3]. This can be the case, for instance, when epidemiologists estimate indicators based on the relative disease incidence or other methods during humanitarian crises [4-6].

Recent epidemiological studies have explored the use and relevance of cell phone connection (CPC) data. Most of these studies model population mobility during various types of crises using CPC data instead of counting the local population [7-9]. In non-emergency settings, seasonal migration can be associated with short-term – and, in some cases, massive – population shifts related to pastoralism or holidays such as the Lunar New Year in China [10-12]. Summer holidays in European countries lead to more modest but still substantial short-term changes in population denominators at a fine geographical scale [13]. These seasonal changes likely cause rapid changes in incidence rate estimates [14]. Documenting population mobility requires fine-scale, complex and time-consuming modelling techniques. However, CPC data may be a more straightforward and accessible means to provide exhaustive real-time estimates of population denominators at a given time in a given area [15].

With a total population of over 12.29 million residents in 2019, the Île-de-France (IdF) Région includes Paris and seven surrounding Départements [16]. Its population density is high, at 1,022 inhabitants per km^2^. Furthermore, IdF remains a prime tourist destination, with 47.5 million visitors in 2023 [17]. On account of its population size, density and mobility, IdF is often the first area of the country affected by large-scale epidemics, i.e. where the highest estimated severe acute respiratory syndrome coronavirus 2 (SARS-CoV-2) seroprevalence rates in France in March–May 2020 was noted [18]. The population is known to vary seasonally: during the summer or winter school holidays, 30% to 45% of the IdF population is estimated to travel to other French Régions or abroad [19]. As epidemiological rates and trends pertain to the at-risk IdF population, which is large and very dense, the resulting public health impact may be substantially underestimated.

Given the uncertainties regarding the actual number of IdF residents actually present in IdF during July–August 2021, the IdF Regional Unit of Santé publique France (SpF, the French National Public Health Agency) computed the incidence data of positive tests for SARS-CoV-2 in IdF using CPC data to estimate the population denominators. The aim of this proof-of-concept exercise was to determine whether CPC data would better describe seasonal population dynamics and better represent denominators for the SARS-CoV-2 epidemic in IdF during the summer of 2021. A secondary aim was to analyse SARS-CoV-2 trends using the effective reproduction number (R_eff_).

## Methods

### Study design and setting

We conducted a longitudinal study to compare the incidence rate of positive SARS-CoV-2 rates among IdF residents published by SpF using fixed population denominators vs rates computed using population estimates derived from CPC data. 

### Commonly computed SARS-CoV-2 census-based incidence rate 

The information system for population-based testing (SIDEP, Système d’Information de Dépistage Populationnel) was an ad hoc nationwide platform used to document all SARS-CoV-2 test results in all French public and private laboratories from May 2020 through June 2023 [20]. Individual PCR or antigenic test results were systematically entered by testing laboratories along with individuals’ Département of residence and place of testing. At the peak of the COVID-19 pandemic, SpF produced mean SARS-CoV-2 incidence estimates per moving week on a daily basis but only referenced by the Département of residence. Thus, positive test results in IdF residents on vacation elsewhere in France were allocated to IdF. This SpF indicator (SpF*i*) was therefore based on the daily number of positive test results for IdF residents according to SIDEP, regardless of where they were tested in France. The denominator used was the 2019 census estimate for the IdF population. The SpF*i* indicator was calculated as follows:


$$SpFi=\left(\frac{Mean\;SARS–CoV–2\;cases\;per\;moving\;week\;in\;IdF\;residents\;in\;all\;Régions}{Fixed\;IdF\;population\;denominator\;of\;12.29\;million}\right)*100,000$$

The threshold of 50 positive cases per 100,000 population was a governmental alert threshold for certain COVID-19 restrictions, leading to the prohibition of informal gatherings with more than 30 persons. The threshold of 250 positive cases per 100,000 was the maximum alert threshold, triggering further control measures such as public venue closures and patient prioritisation plans in hospitals.

Using SIDEP, we extracted and deduplicated the daily number of positive PCR and antigenic SARS-CoV-2 tests performed on IdF residents of all ages who were only tested in IdF laboratories between 28 June and 9 September 2021. The R_eff_ for positive test results was modelled using the EpiEstim method (R software). We used a serial interval of 8.4 (standard deviation (SD): 3.8) computed by the Markov chain Monte Carlo method used in the R package [21].

### FluxVision cell phone connection data

We obtained denominator estimates through FluxVision, a dataset proposed by Orange (the main cell phone service provider in France) that measures the number of people who connect to all geolocated terminals in IdF at midnight [9,15,22,23]. We did not count mobile phone connections directly but used data from a major telecom provider that models population estimates based on phone usage patterns. While we lack access to the proprietary modelling algorithms, the method relies on market share, age, origin, and average people-per-phone ratios to estimate the population present in a given time and place of interest [23]. FluxVision results therefore estimate the total number of people of all ages present in an area and not only Orange cell phone network subscribers. The data differentiate between full-time residents, residents ‘in transit’ (travel), tourists, excursionists (day trippers), repeat excursionists, habitual visitors, habitual visitors in transit, and people simply in transit. A description of the categories and numbers of individuals present in the FluxVision database are provided in Supplementary Table S1 and Supplementary Figure S1. These data were provided to us free of charge for the IdF Région for the purpose of the study, without specific details about the Département. The population estimates are aggregated and thus strictly anonymous to protect privacy [24].

### Real-time IdF population denominator and corrected incidence rate

We only considered IdF residents, which represented 67% of the population present in the FluxVision database of modelled daily estimates (provided in Supplementary Table S1). This was based on the assumption that tourists and excursionists had a lower probability of undergoing PCR tests, vaccination, and health-seeking behaviours for a given day spent in IdF. In 2021, tourists remained for an average of 2.3–2.5 days in IdF hotels, thus reducing the risk of contributing notably to the circulation of SARS-CoV-2 in IdF [25]. 

**Figure 1:**
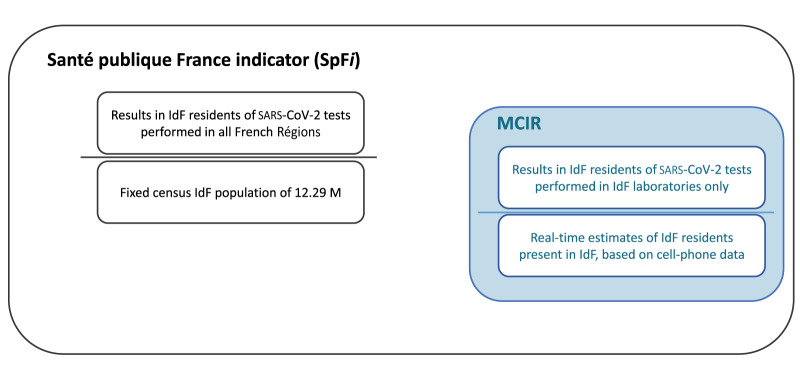
Overview of mean official (SpF*i*) incidence rate per moving week and mean corrected incidence rate (MCIR) per moving week, Île-de-France, 4 July–9 September 2021

IdF: Île-de-France; MCIR: mean corrected incidence rate; SpF*i*: Santé publique France (SpF) published incidence; SARS-CoV-2: severe acute respiratory syndrome coronavirus 2.

To compute the mean corrected incidence rate (MCIR) per moving week between 4 July and 9 September 2021 (1 week after schools reopened), we used the daily number of positive tests (of IdF residents) actually carried out in IdF laboratories as the numerator and the estimated resident population actually present in IdF at midnight on that same day as the denominator (Figure 1).


$$MCIR=\left(\frac{Mean\;number\;of\;SARS–CoV–2\;cases\;per\;moving\;week\;in\;IdF\;residents\;present\;in\;IdF}{Estimated\;number\;of\;IdF\;residents\;present\;based\;on\;cell\;phone\;connections}\right)*100,000$$

This MCIR for each moving week was compared with the daily incidence rate published by SpF, presented in a table and plotted in a line graph.

### Sensitivity analysis

The corrected MCIR was recalculated using all positive tests carried out in IdF laboratories as the numerator based on SIDEP (including both IdF and non-IdF residents) and the population of IdF residents as the denominator but adding the category of ‘habitual visitors’ (more likely to adopt similar screening behaviours). These people did not reside in IdF but stayed there on a regular basis and had spent more than 2 h in IdF in the previous 24 h.

## Results

### Changes in the computed SARS-CoV-2 incidence rate

**Figure 2:**
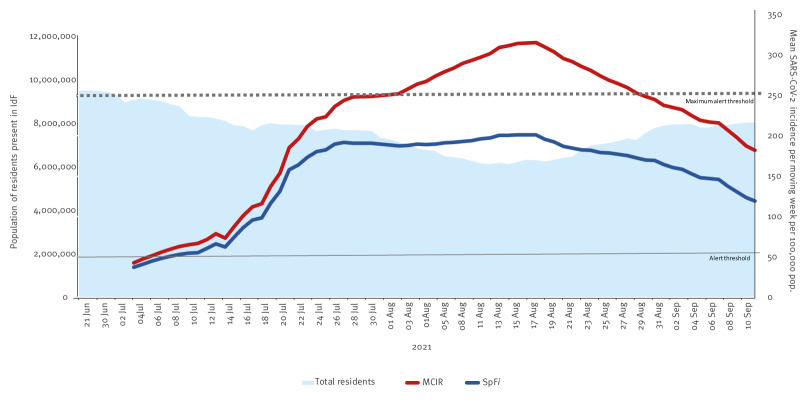
SpF and corrected SARS-CoV-2 incidence by moving week, Île-de-France, 28 June–9 September 2021^a^

IdF: Île-de-France; MCIR: mean corrected incidence rate; SARS-CoV-2: severe acute respiratory coronavirus 2; SpF: Santé publique France; SpF*i*: SARS-CoV-2 incidence by moving week computed by SpF.

^a^ Data from 28 June–9 September were used for computations. The moving one-week incidence can only be computed starting 4 July.

The corrected (MCIR) values differed from the mean weekly SpF*i* per rolling week based on fixed population denominators. These negative or positive differences began at the start of the study period and varied depending on the date and seasonal mobility (Table and Figure 2).

**Table:** Numbers and corrected SARS-CoV-2 incidence rates of Île-de-France residents, 28 June–9 September 2021

Date 2021	Number of positive SARS-CoV-2 tests^a^			Residents in the FluxVision database	Daily corrected incidence rate per 100,000 population	Mean incidence per 100,000 population by moving week		
	IdF residents tested in IdF	IdF cases among IdF residents according to SpF	Δ			Corrected incidence	Official incidence	Correction factor
28 Jun	625	700	75	9,368,315	5.11	NA	NA	NA
29 Jun	567	599	32	9,417,743	4.62	NA	NA	NA
30 Jun	620	729	109	9,402,904	5.06	NA	NA	NA
1 Jul	658	734	76	9,354,217	5.39	NA	NA	NA
2 Jul	810	930	120	9,233,474	6.71	NA	NA	NA
3 Jul	496	614	118	8,791,364	4.29	NA	NA	NA
4 Jul	203	260	57	8,890,363	1.73	42.6	37.2	1.15
5 Jul	1,065	1,208	143	9,030,063	9.01	47.6	41.3	1.15
6 Jul	922	1,023	101	8,984,674	7.86	51.7	44.7	1.16
7 Jul	975	1,153	178	8,867,867	8.36	56.0	48.2	1.16
8 Jul	956	1,108	152	8,763,914	8.34	59.8	51.2	1.17
9 Jul	1,047	1,169	122	8,653,650	9.23	63.0	53.2	1.18
10 Jul	644	842	198	8,145,870	5.98	65.2	55.0	1.19
11 Jul	266	328	62	8,093,758	2.48	66.8	55.6	1.20
12 Jul	1,435	1,830	395	8,162,318	13.42	72.0	60.7	1.19
13 Jul	1,409	1,724	315	8,103,802	13.30	78.8	66.4	1.19
14 Jul	430	642	212	7,940,343	4.10	73.5	62.2	1.18
15 Jul	2,078	2,707	629	7,790,624	20.33	88.3	75.1	1.18
16 Jul	2,009	2,556	547	7,742,682	19.78	101.5	86.3	1.18
17 Jul	1,398	1,980	582	7,472,119	14.12	112.1	95.6	1.17
18 Jul	501	709	208	7,654,257	4.90	115.8	98.7	1.17
19 Jul	3,085	4,023	938	7,866,306	29.86	137.1	116.5	1.18
20 Jul	2,668	3,522	854	7,849,598	25.98	153.6	131.2	1.17
21 Jul	2,912	3,887	975	7,850,977	28.36	185.5	157.8	1.18
22 Jul	2,954	3,487	533	7,828,401	28.85	196.5	164.1	1.20
23 Jul	3,197	3,726	529	7,781,720	31.31	211.4	173.6	1.22
24 Jul	2,116	2,843	727	7,470,846	21.43	220.6	180.6	1.22
25 Jul	672	948	276	7,504,397	6.75	223.6	182.6	1.22
26 Jul	4,008	4,878	870	7,628,696	40.03	236.4	189.5	1.25
27 Jul	3,158	3,779	621	7,586,028	31.83	243.8	191.7	1.27
28 Jul	3,172	3,764	592	7,576,108	32.01	248.4	190.6	1.30
29 Jul	2,872	3,505	633	7,492,714	29.11	248.7	190.7	1.30
30 Jul	3,096	3,611	515	7,126,609	32.67	250.0	189.7	1.32
31 Jul	2,037	2,674	637	7,037,423	21.76	250.8	188.4	1.33
1 Aug	559	809	250	6,987,336	6.07	252.1	187.3	1.35
2 Aug	4,165	4,988	823	6,849,271	46.37	258.1	188.2	1.37
3 Aug	3,288	3,965	677	6,739,074	37.21	264.2	189.7	1.39
4 Aug	3,049	3,692	643	6,672,172	34.83	267.2	189.2	1.41
5 Aug	2,979	3,604	625	6,609,530	34.24	273.6	190.0	1.44
6 Aug	3,141	3,758	617	6,285,241	37.54	279.2	191.1	1.46
7 Aug	2,018	2,754	736	6,258,534	24.26	283.7	191.8	1.48
8 Aug	653	928	275	6,248,999	7.91	289.5	192.8	1.50
9 Aug	4,137	5,176	1,039	6,126,505	51.17	293.5	194.3	1.51
10 Aug	3,259	4,198	939	6,046,044	40.99	297.5	196.2	1.52
11 Aug	3,048	3,835	787	6,013,418	38.51	301.8	197.4	1.53
12 Aug	3,206	4,003	797	6,046,473	40.18	309.2	200.7	1.54
13 Aug	3,108	3,766	658	5,921,541	39.37	311.3	200.7	1.55
14 Aug	2,095	2,822	727	6,025,149	26.09	314.2	201.3	1.56
15 Aug	676	942	266	6,198,942	8.27	315.0	201.4	1.56
16 Aug	4,191	5,123	932	6,182,403	51.50	315.6	200.9	1.57
17 Aug	2,959	3,569	610	6,140,664	36.64	310.0	195.9	1.58
18 Aug	2,758	3,413	655	6,209,404	33.81	304.1	192.4	1.58
19 Aug	2,767	3,312	545	6,271,557	33.48	295.5	186.8	1.58
20 Aug	3,021	3,507	486	6,294,568	36.16	291.8	184.7	1.58
21 Aug	1,953	2,543	590	6,588,950	22.31	285.9	182.4	1.57
22 Aug	597	908	311	6,852,504	6.63	280.7	182.2	1.54
23 Aug	4,075	4,754	679	6,937,917	44.86	274.5	179.2	1.53
24 Aug	2,906	3,502	596	6,995,676	31.73	268.8	178.7	1.50
25 Aug	2,815	3,215	400	7,078,949	30.36	264.8	177.1	1.50
26 Aug	2,742	3,125	383	7,169,753	29.12	259.6	175.5	1.48
27 Aug	2,855	3,194	339	7,070,879	30.58	253.3	173.0	1.46
28 Aug	1,898	2,205	307	7,387,111	19.45	248.6	170.2	1.46
29 Aug	607	806	199	7,690,947	6.03	244.7	169.4	1.44
30 Aug	3,878	4,141	263	7,813,409	38.04	238.1	164.4	1.45
31 Aug	2,973	3,061	88	7,870,580	28.96	235.1	160.8	1.46
1 Sep	2,870	2,937	67	7,878,964	27.94	232.4	158.6	1.47
2 Sep	2,407	2,483	76	7,883,382	23.40	225.2	153.3	1.47
3 Sep	2,557	2,594	37	7,681,671	25.37	219.1	148.4	1.48
4 Sep	1,800	2,057	257	7,669,081	17.86	216.8	147.2	1.47
5 Sep	555	644	89	7,820,919	5.43	215.7	145.9	1.48
6 Sep	3,218	3,137	−81	7,864,180	31.41	207.2	137.7	1.50
7 Sep	2,241	2,199	−42	7,905,819	21.74	197.8	130.7	1.51
8 Sep	2,096	2,049	−47	7,957,984	20.21	187.8	123.5	1.52
9 Sep	1,977	1,939	−38	7,973,448	19.00	182.0	119.1	1.53

IdF: Île-de-France; NA: not applicable, the mean incidences being computed by moving week; SARS-CoV-2: severe acute respiratory coronavirus 2.

^a^ Positive SARS-CoV-2 tests include both PCR + antigenic tests.

The alert threshold at 50 positive cases per 100,000 population was a governmental alert threshold for certain COVID-19 restrictions, leading to the prohibition of informal gatherings with more than 30 persons. The threshold of 250 positive cases per 100,000 was the maximum alert threshold, triggering further control measures such as public venue closures and patient prioritisation plans in hospitals.

According to the epidemic curve, the MCIR values were initially higher than the published estimates, showing that the circulation of SARS-CoV-2 virus among those present in IdF was more intense than estimated by SpF. This upward shift was particularly marked during the entire month of August, reaching a peak on 16 August when the SpF*i* rate per moving week was 200.9 per 100,000 compared with 315.6 per 100,000 for the MCIR, a difference of + 57%. The SpF*i* per moving week remained well below the maximum alert threshold value of 250 per 100,000, although the MCIR surpassed this threshold from 30 July–27 August 2021 (29 days).

### Changes in the denominator

Changes in the denominator were largely attributable to population movements during the summer period. For example, on 17 August, the population of IdF residents present in IdF was estimated at only 6,140,664 instead of the fixed estimate of 12,291,600, a difference of 6,150,936 inhabitants (−50.0%). These differences also affected the number of positive tests carried out on IdF residents (numerator). Indeed, IdF residents (regardless of their location) had 3,569 positive tests on 17 August according to SpF, whereas only 2,959 of these tests were carried out in IdF laboratories on IdF residents, a difference of 610 positive tests (20.6% lower than SpF data). At the start of the new school year in September coinciding with the end of the study period, the MCIR and SpF*i* rates had still not reconverged.

### Estimated effective R

**Figure 3:**
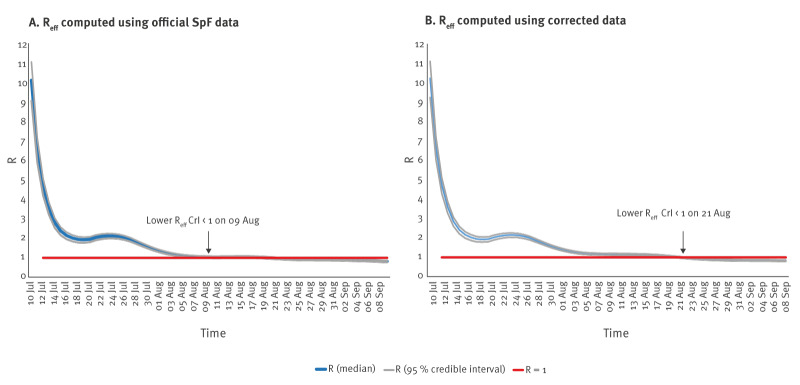
Impact of discrepancies between official SpF data (A) and corrected data (B) on estimated real-time R_eff_ values of SARS-CoV-2 cases among Île-de-France residents, 28 June–9 September 2021^a^

CrI: credible interval; R_eff_ : effective reproduction numbers; SARS-CoV-2: severe acute respiratory coronavirus 2; SpF: Santé publique France.

^a^ Data from 28 June–9 September were used for computations.

Between 10 July and 9 September, the mean weekly moving R_eff_ using corrected data was higher than the mean R_eff_ computed using the official SpF data for a duration of 39 days (Figure 3) with the difference varying between 1% and 12%. The effective R estimates and credible intervals (CI) for uncorrected SpF*i* and corrected MCIR among residents and habitual visitors are provided in Supplementary Table S2. The corrected R_eff_ was significantly above 1 for 42 days (until August 21) compared with 29 days for the R_eff_ computed using SpF data (until August 9) during the study period.

Effective reproduction numbers and trends computed using (A) official SpF data on positive SARS-CoV-2 tests in Île-de-France residents wherever they were tested throughout France and (B) corrected data on positive SARS-CoV-2 tests in Île-de-France residents tested in Île-de-France laboratories.

### Sensitivity analysis

Sensitivity analysis (see Supplementary Table S3 and Supplementary Figure S2 for numbers and corrected incidence rates, and graphical representation, respectively) relating to the resident populations as well as habitual visitors (defined in Supplementary Table S1) revealed a greater difference between SpF*i* and MCIR compared with data relating only to IdF residents present in IdF during the study period. At its lowest level on 11 August, the population present in IdF represented 50.8% of the maximum resident population during the period (with 3,048 tests carried out in IdF on IdF residents) compared with 54.3% of the maximum population based on residents and habitual visitors (with 3,442 tests carried out in IdF). However, the MCIR values based on IdF residents and habitual visitors remained above the maximum alert threshold for a period of 31 days.

## Discussion

Population density is a key driving force behind communicable disease epidemics, especially for contagious diseases such as COVID-19. Seasonal and other mobility patterns affect the risk of disease propagation between different areas [26]. Mobility patterns also impact the risk of disease transmission between individuals in a given area as well as the epidemiological estimation of attack or incidence rates [27]. Rates reported by SpF at the scale of mainland France are sufficiently accurate throughout the year. However, we can markedly improve the accuracy of SpF*i* rates reported at the Région and especially Département levels during vacation periods.

Our fine-scale estimates of population denominators using CPC data are particularly relevant in small geographical areas with highly dense and mobile populations as in the case of IdF. The summertime migration of IdF residents is well documented [13] and reflected in our 2021 CPC data. According to Google mobility data, the lowest number of residents was present in IdF in early to mid-August 2021, with variations at the Département level [28].

Our corrected MCIR showed a higher and longer peak than that of SpF*i* during the summer of 2021. In addition to the more severe SARS-CoV-2 Delta variant [29], which was the majority strain during the summer of 2021 [30], this discrepancy may have somewhat distorted predictions of critical care bed occupancy rates (STEP tool in IdF; data not shown). It also affected R_eff_ modelling estimates for the region, with a maximum variation of ca 10%.

Although the SpF*i* estimates are correct and simple to compute, they pertain to all IdF residents regardless of their location in France. Our method provides more accurate data about those individuals actually present locally, who may benefit from local prevention campaigns or healthcare resources.

Very few fixed disease surveillance thresholds determine specific public health interventions, aside from meningitis [31]. From an epidemiological perspective, incidence rates are of modest importance per se given surveillance biases, minimally symptomatic or asymptomatic SARS-CoV-2 infections, different screening practices and underreporting. Epidemiologists mostly base their risk assessments on trends. However, this is not the case for decisionmakers: government thresholds of 50 and 250 positive cases per 100,000 population led to alerts and maximum alerts, respectively, with different control measures. During the study period, the MCIR using the estimated resident population data crossed the maximum alert threshold of 250 cases per 100,000, although the SpF*i* indicator did not. Regardless of whether habitual visitors were included, the MCIR crossed the maximum alert threshold for a duration of at least 29 days. The high vaccination coverage and natural immunisation rates in IdF achieved by the summer of 2021, which was not affected by incidence estimates, fortunately mitigated the impact of severe cases on healthcare structures and slowed down the circulation of the SARS-CoV-2 virus. Had these higher incidence rates been known at the time, however, countries may have restricted travel to and from IdF during that period.

Our study has several strengths. It is a longitudinal analysis based on a very high number of tests and an exhaustive estimate of a population comparable to that of a middle-sized European country. Assumptions and data sources are well corroborated, and the test results were exhaustively documented with over 150,000 positive test results thanks to the SIDEP system.

Our study also has some limitations. Firstly, cell phone ownership is variable, especially in low-income countries. Wesolowski et al. showed in 2013 in Kenya that mobility and connection estimates are robust if an adjustment scheme allowing for differential ownership is applied, as in FluxVision’s modelling of population estimates [15,23]. In mid-2021, IdF had a high density of antennae, with cell phone ownership exceeding 94% [32]. We also examined corrected incidence rates over a short period of time, thereby reducing the risk of bias because of changes in cell phone ownership and antenna distribution. Secondly, there is some ‘black box’ effect, as we did not have access to FluxVision’s commercially protected algorithms and scripts. These available population estimates have nevertheless been used for a decade in the tourism industry and found to be robust when compared with hotel stays [33]. They have also been used by modellers [9]. Thirdly, it is not certain whether habitual visitors in IdF would adopt the same behaviour as IdF residents, especially in terms of screening. According to FluxVision, habitual visitors and residents have similar behaviour, unlike other categories such as tourists and day-trippers. Habitual visitors represent a relatively modest 7% of the database compared with 68% for residents, making up a total of 75% (Supplementary Table S1). Excluding tourists and day-trippers may have overestimated the MCIR as these individuals, however short their stay, probably have lower risks of acquiring or transmitting SARS-CoV-2 in the community and healthy travellers may be more often pauci- or asymptomatic. Fourthly, we lack data on residents and habitual visitors stratified by age group. For example, elderly (aged ≥ ­65 years) or dependent people are comparatively less likely to travel outside their region of residence [34]. The incidence rate may therefore have been over- or underestimated, depending on the age group considered. Fifthly, the MCIR computed for IdF residents who tested positive in IdF during summer may have overrepresented IdF residents who did not or could not go on holiday, thus overrepresenting essential workers and socially disadvantaged families living in high-density housing, all factors known to be associated with SARS-CoV-2 infection [9,35]. Our study, however, aimed to measure the MCIR in real time in the majority of the population actually present in IdF during summer when other IdF residents travelled elsewhere for holidays; we did not seek to measure the average rate across all IdF residents wherever they were. Sixthly, we only had access to cell phone data at the Régional level, not at the Départemental level. The IdF Région includes both the richest (Hauts-de-Seine) and the poorest Département of mainland France (Seine-Saint-Denis). The gap between the SpF*i* calculated by SpF using fixed denominators and the MCIR may have varied greatly depending on the Département and the proportion of vacationers in their population, as shown by Google Mobility data. Once again, our study aimed to measure the overall MCIR among those present in the IdF Région during the summer of 2021.

## Conclusion

Using local testing data and real-time population denominators based on CPC data, we showed that indicators computed using non-geographically referenced test results and fixed population denominators that ignore seasonal mobility can markedly underestimate incidence rates in IdF during the summer holidays. Lessons were learned regarding the reliable estimation of the incidence rates of SARS-CoV-2 and other infections during holiday periods in the regions of mainland France as well as abroad. New data sources based on CPC data offer the opportunity to calculate much more accurate and dynamic incidence rates, thus improving documentation and fine-scale mapping of the epidemiological context and dynamic incidence rates to better guide risk assessment and responses. The lessons learned from this proof-of-concept exercise were used for the real-time and fine-scale estimates of the population present during the Paris 2024 Summer Olympic and Paralympic Games.

## Supplementary Data

1220000 Supplement
