# Supplementary material for: Use of cell phone data to correct Île-de-France population estimates and SARS-CoV-2 incidence, July to September, 2021: a proof-of-concept exercise
Source: Euro Surveill. 2025 Jun 5;30(22):2400530. doi: 10.2807/1560-7917.ES.2025.30.22.2400530 (PMC12143120; doi:10.2807/1560-7917.ES.2025.30.22.2400530)
Supplement: Supplement [file 24-00530_TARANTOLA_Supplement.pdf]

This supplementary material is hosted by *Eurosurveillance* as supporting information alongside the article "Use of cell phone data to correct Île-de-France population estimates and SARS-CoV-2 incidence, July to September, 2021: a proof-of-concept exercise" on behalf of the authors who remain responsible for the accuracy and appropriateness of the content. The same standards for ethics, copyright, attributions and permissions as for the article apply. *Eurosurveillance* is not responsible for the maintenance of any links or email addresses provided therein.

SUPPLEMENTARY TABLE S1: Categories and numbers of persons present in the FluxVision database, June 28 to September 9, 2021, Île-de-France.

Categories	Description	Person-days	% of total
Present for 2 hours or more in Île-de-France			
Resident	Resident spending a night in the observed area, present for more than 2 hours, and at least 22 nights (consecutive or otherwise) in the previous 56-day moving time period	554,683,092	67%
Habitual visitor	Non-resident but often present and spending a night in the observed area, present for more than 2 hours and more than four times in different weeks out of the previous 56-day moving time period	54,190,648	7%
Tourist	Non-resident, not usually present, and spending a night in the observed area, present for more than 2 hours and less than four times in different weeks out of the previous 56-day moving time period	77,650,360	9%
Excursionist (Day-tripper)	Non-resident, not habitually present and not spending the previous night or present evening in the study area and present less than five times during the day during the previous 15 days.	28,693,001	3%
Recurring day-tripper	Person not spending the previous night or current evening in the study area and present five times or more during the day during the previous 15 days.	50,899,516	6%
Present less than 2 hours in the study area			
Resident, in transit	Resident not spending the previous or current night in the area and present less than 2 hours in the day during that particular day.	9,879,059	1%
Habitual visitor, in transit	Person often present, not spending the previous or current night in the area and present less than 2 hours during that particular day.	2,141,997	0%
Tourist, in transit	Tourist, not spending the previous or current night in the area and present less than 2 hours in the day during that particular day.	5,875,938	1%
Transit	Other person present for less than 2 hours in the area	38,871,329	5%
**Total**		**822,884,940**	**100%**

SUPPLEMENTARY TABLE S2: Effective R estimates and credible intervals (CI), taking into consideration uncorrected SpFi and corrected MCIR among residents and habitual visitors, Île-de-France, June 28 to September 9, 2021.

Time period (7 days)		R_{eff} of uncorrected SpF*i* incidence			R_{eff} of MCIR (Residents)			R_{eff} of MCIR (Resident + habitual visitors)		
START	END	Mean R	2.5% CI	97.5% CI	Mean R	2.5 % CI	97.5% CI	Mean R	2.5% CI	97.5% CI
4 Jul	10 Jul	10.22	9.15	11.16	10.30	9.30	11.17	10.33	9.36	11.17
5 Jul	11 Jul	6.64	5.96	7.23	6.71	6.08	7.26	6.71	6.10	7.24
6 Jul	12 Jul	4.70	4.24	5.11	4.76	4.32	5.14	4.74	4.32	5.11
7 Jul	13 Jul	3.57	3.23	3.88	3.63	3.30	3.91	3.61	3.30	3.88
8 Jul	14 Jul	2.82	2.56	3.06	2.87	2.62	3.09	2.86	2.62	3.07
9 Jul	15 Jul	2.40	2.18	2.59	2.43	2.23	2.61	2.43	2.23	2.60
10 Jul	16 Jul	2.16	1.97	2.33	2.18	2.00	2.34	2.20	2.02	2.35
11 Jul	17 Jul	2.03	1.86	2.18	2.04	1.88	2.18	2.07	1.91	2.20
12 Jul	18 Jul	1.95	1.79	2.09	1.95	1.81	2.08	1.99	1.84	2.11
13 Jul	19 Jul	1.94	1.79	2.07	1.94	1.80	2.06	1.98	1.84	2.10
14 Jul	20 Jul	1.96	1.81	2.08	1.95	1.82	2.07	2.00	1.86	2.11
15 Jul	21 Jul	2.06	1.91	2.18	2.04	1.91	2.16	2.09	1.96	2.20
16 Jul	22 Jul	2.11	1.97	2.23	2.11	1.98	2.22	2.15	2.02	2.26
17 Jul	23 Jul	2.14	2.01	2.26	2.15	2.03	2.26	2.18	2.06	2.28
18 Jul	24 Jul	2.14	2.01	2.25	2.16	2.05	2.27	2.18	2.07	2.28
19 Jul	25 Jul	2.11	1.99	2.21	2.15	2.03	2.25	2.16	2.05	2.25
20 Jul	26 Jul	2.04	1.93	2.14	2.10	1.99	2.19	2.09	1.99	2.18
21 Jul	27 Jul	1.95	1.84	2.04	2.02	1.92	2.10	2.01	1.91	2.09
22 Jul	28 Jul	1.82	1.72	1.90	1.90	1.81	1.98	1.88	1.79	1.96
23 Jul	29 Jul	1.69	1.60	1.77	1.78	1.70	1.86	1.75	1.67	1.82
24 Jul	30 Jul	1.56	1.48	1.64	1.66	1.59	1.73	1.63	1.55	1.69
25 Jul	31 Jul	1.45	1.37	1.52	1.55	1.48	1.62	1.51	1.44	1.57
26 Jul	1 Aug	1.35	1.28	1.42	1.46	1.39	1.52	1.41	1.34	1.46
27 Jul	2 Aug	1.27	1.20	1.33	1.38	1.32	1.44	1.32	1.26	1.37
28 Jul	3 Aug	1.20	1.14	1.26	1.31	1.25	1.37	1.26	1.20	1.30
29 Jul	4 Aug	1.15	1.09	1.20	1.26	1.20	1.31	1.20	1.15	1.25
30 Jul	5 Aug	1.11	1.05	1.16	1.22	1.17	1.27	1.17	1.12	1.21
31 Jul	6 Aug	1.08	1.02	1.13	1.20	1.14	1.24	1.15	1.10	1.19
1 Aug	7 Aug	1.06	1.01	1.11	1.18	1.13	1.23	1.14	1.09	1.18
2 Aug	8 Aug	1.05	1.00	1.10	1.17	1.12	1.22	1.14	1.09	1.18
3 Aug	9 Aug	1.04	0.99	1.09	1.17	1.11	1.21	1.14	1.09	1.18
4 Aug	10 Aug	1.04	0.98	1.09	1.16	1.11	1.20	1.14	1.09	1.18
5 Aug	11 Aug	1.04	0.98	1.08	1.16	1.11	1.20	1.14	1.09	1.18
6 Aug	12 Aug	1.04	0.99	1.09	1.16	1.11	1.20	1.14	1.10	1.19
7 Aug	13 Aug	1.04	0.99	1.09	1.15	1.10	1.20	1.15	1.10	1.19
8 Aug	14 Aug	1.05	0.99	1.09	1.15	1.10	1.19	1.15	1.10	1.19
9 Aug	15 Aug	1.05	0.99	1.09	1.14	1.10	1.18	1.15	1.10	1.19

10 Aug	16 Aug	1.05	0.99	1.09	1.14	1.09	1.18	1.14	1.09	1.18
11 Aug	17 Aug	1.04	0.99	1.09	1.12	1.08	1.16	1.12	1.08	1.16
12 Aug	18 Aug	1.03	0.98	1.08	1.11	1.06	1.14	1.10	1.06	1.14
13 Aug	19 Aug	1.02	0.96	1.06	1.08	1.04	1.12	1.08	1.03	1.11
14 Aug	20 Aug	1.00	0.95	1.05	1.06	1.01	1.09	1.04	1.00	1.08
15 Aug	21 Aug	0.98	0.93	1.03	1.03	0.99	1.07	1.01	0.97	1.05
16 Aug	22 Aug	0.96	0.91	1.01	1.00	0.96	1.04	0.98	0.94	1.01
17 Aug	23 Aug	0.95	0.90	0.99	0.97	0.93	1.01	0.94	0.90	0.98
18 Aug	24 Aug	0.94	0.88	0.98	0.95	0.91	0.98	0.91	0.87	0.95
19 Aug	25 Aug	0.93	0.88	0.97	0.93	0.89	0.96	0.89	0.85	0.92
20 Aug	26 Aug	0.92	0.87	0.97	0.91	0.87	0.94	0.87	0.83	0.90
21 Aug	27 Aug	0.92	0.87	0.96	0.89	0.85	0.93	0.85	0.81	0.88
22 Aug	28 Aug	0.92	0.87	0.96	0.88	0.84	0.92	0.84	0.80	0.87
23 Aug	29 Aug	0.92	0.87	0.96	0.87	0.83	0.91	0.83	0.79	0.86
24 Aug	30 Aug	0.91	0.86	0.96	0.87	0.83	0.90	0.82	0.78	0.85
25 Aug	31 Aug	0.91	0.86	0.95	0.86	0.82	0.89	0.81	0.78	0.85
26 Aug	1 Sep	0.91	0.85	0.95	0.86	0.82	0.89	0.81	0.77	0.84
27 Aug	2 Sep	0.90	0.85	0.94	0.85	0.81	0.89	0.81	0.77	0.84
28 Aug	3 Sep	0.89	0.84	0.93	0.85	0.81	0.88	0.80	0.76	0.84
29 Aug	4 Sep	0.88	0.83	0.92	0.85	0.81	0.88	0.80	0.76	0.84
30 Aug	5 Sep	0.87	0.82	0.92	0.85	0.81	0.88	0.80	0.76	0.83
31 Aug	6 Sep	0.86	0.81	0.91	0.85	0.81	0.88	0.80	0.76	0.83
1 Sep	7 Sep	0.85	0.80	0.89	0.84	0.80	0.88	0.79	0.75	0.83
2 Sep	8 Sep	0.83	0.78	0.88	0.83	0.79	0.87	0.79	0.75	0.82
3 Sep	9 Sep	0.82	0.76	0.86	0.83	0.78	0.86	0.78	0.74	0.82

SUPPLEMENTARY TABLE S3. Numbers and corrected SARS-CoV-2 incidence rates of Île-de-France residents and habitual visitors, 28 June–9 September 2021

Date 2021	Number of positive SARS-CoV-2 tests			Residents + habitual visitors in the FluxVision database	Daily corrected incidence rate per 100,000 population	Mean incidence per 100,000 population by moving week		
	IdF residents tested in IdF	IdF cases among IdF residents according to SpF	Δ			Corrected incidence	Official incidence	Correction factor
28 Jun	714	700	-14	10,228,738	6.98	NA	NA	NA
29 Jun	653	599	-54	10,260,708	6.36	NA	NA	NA
30 Jun	736	729	-7	10,213,051	7.21	NA	NA	NA
1 Jul	766	734	-32	10,173,919	7.53	NA	NA	NA
2 Jul	927	930	3	10,153,004	9.13	NA	NA	NA
3 Jul	602	614	12	9,745,434	6.18	NA	NA	NA
4 Jul	256	260	4	9,761,178	2.62	46.0	37.2	1.24
5 Jul	1,227	1,208	-19	9,815,496	12.50	51.5	41.3	1.25
6 Jul	1,065	1,023	-42	9,768,251	10.90	56.1	44.7	1.25
7 Jul	1,146	1,153	7	9,721,712	11.79	60.7	48.2	1.26
8 Jul	1,094	1,108	14	9,549,717	11.46	64.6	51.2	1.26
9 Jul	1,166	1,169	3	9,491,104	12.29	67.7	53.2	1.27
10 Jul	779	842	63	9,012,644	8.64	70.2	55.0	1.28
11 Jul	318	328	10	8,922,750	3.56	71.1	55.6	1.28
12 Jul	1,665	1,830	165	8,910,431	18.69	77.3	60.7	1.27
13 Jul	1,649	1,724	75	8,807,703	18.72	85.1	66.4	1.28
14 Jul	550	642	92	8,670,093	6.34	79.7	62.2	1.28
15 Jul	2,446	2,707	261	8,441,554	28.98	97.2	75.1	1.29
16 Jul	2,340	2,556	216	8,454,438	27.68	112.6	86.3	1.30
17 Jul	1,653	1,980	327	8,231,149	20.08	124.1	95.6	1.30
18 Jul	635	709	74	8,442,542	7.52	128.0	98.7	1.30
19 Jul	3,645	4,023	378	8,602,196	42.37	151.7	116.5	1.30
20 Jul	3,151	3,522	371	8,550,928	36.85	169.8	131.2	1.29
21 Jul	3,464	3,887	423	8,542,327	40.55	204.0	157.8	1.29
22 Jul	3,543	3,487	-56	8,516,602	41.60	216.7	164.1	1.32
23 Jul	3,707	3,726	19	8,523,550	43.49	232.5	173.6	1.34
24 Jul	2,420	2,843	423	8,228,994	29.41	241.8	180.6	1.34
25 Jul	844	948	104	8,237,992	10.25	244.5	182.6	1.34
26 Jul	4,566	4,878	312	8,321,063	54.87	257.0	189.5	1.36
27 Jul	3,529	3,779	250	8,280,303	42.62	262.8	191.7	1.37
28 Jul	3,523	3,764	241	8,260,213	42.65	264.9	190.6	1.39
29 Jul	3,162	3,505	343	8,253,567	38.31	261.6	190.7	1.37
30 Jul	3,375	3,611	236	7,906,117	42.69	260.8	189.7	1.37
31 Jul	2,232	2,674	442	7,787,111	28.66	260.0	188.4	1.38
1 Aug	705	809	104	7,665,375	9.20	259.0	187.3	1.38
2 Aug	4,608	4,988	380	7,491,525	61.51	265.6	188.2	1.41
3 Aug	3,651	3,965	314	7,412,578	49.25	272.3	189.7	1.43
4 Aug	3,408	3,692	284	7,328,566	46.50	276.1	189.2	1.46
5 Aug	3,242	3,604	362	7,301,272	44.40	282.2	190.0	1.49

6 Aug	3,441	3,758	317	7,007,439	49.10	288.6	191.1	1.51
7 Aug	2,259	2,754	495	6,932,873	32.58	292.6	191.8	1.53
8 Aug	817	928	111	6,911,195	11.82	295.2	192.8	1.53
9 Aug	4,656	5,176	520	6,770,309	68.77	302.4	194.3	1.56
10 Aug	3,686	4,198	512	6,676,204	55.21	308.4	196.2	1.57
11 Aug	3,442	3,835	393	6,697,674	51.39	313.3	197.4	1.59
12 Aug	3,574	4,003	429	6,761,409	52.86	321.7	200.7	1.60
13 Aug	3,406	3,766	360	6,668,244	51.08	323.7	200.7	1.61
14 Aug	2,416	2,822	406	6,755,840	35.76	326.9	201.3	1.62
15 Aug	801	942	141	6,923,023	11.57	326.6	201.4	1.62
16 Aug	4,613	5,123	510	6,902,453	66.83	324.7	200.9	1.62
17 Aug	3,260	3,569	309	6,841,830	47.65	317.1	195.9	1.62
18 Aug	3,033	3,413	380	6,964,891	43.55	309.3	192.4	1.61
19 Aug	3,006	3,312	306	7,171,390	41.92	298.4	186.8	1.60
20 Aug	3,143	3,507	364	7,215,732	43.56	290.8	184.7	1.57
21 Aug	2,161	2,543	382	7,531,425	28.69	283.8	182.4	1.56
22 Aug	752	908	156	7,828,315	9.61	281.8	182.2	1.55
23 Aug	4,292	4,754	462	7,908,802	54.27	269.2	179.2	1.50
24 Aug	3,206	3,502	296	8,012,637	40.01	261.6	178.7	1.46
25 Aug	3,031	3,215	184	8,135,631	37.26	255.3	177.1	1.44
26 Aug	2,957	3,125	168	8,352,490	35.40	248.8	175.5	1.42
27 Aug	3,015	3,194	179	8,405,135	35.87	241.1	173.0	1.39
28 Aug	2,058	2,205	147	8,726,719	23.58	236.0	170.2	1.39
29 Aug	744	806	62	9,065,888	8.21	234.6	169.4	1.38
30 Aug	4,094	4,141	47	9,182,207	44.59	224.9	164.4	1.37
31 Aug	3,096	3,061	-35	9,220,655	33.58	218.5	160.8	1.36
1 Sep	2,957	2,937	-20	9,218,528	32.08	213.3	158.6	1.35
2 Sep	2,544	2,483	-61	9,315,322	27.31	205.2	153.3	1.34
3 Sep	2,672	2,594	-78	9,165,711	29.15	198.5	148.4	1.34
4 Sep	1,938	2,057	119	9,222,514	21.01	195.9	147.2	1.33
5 Sep	631	644	13	9,304,095	6.78	194.5	145.9	1.33
6 Sep	3,306	3,137	-169	9,237,333	35.79	185.7	137.7	1.35
7 Sep	2,255	2,199	-56	9,240,295	24.40	176.5	130.7	1.35
8 Sep	2,086	2,049	-37	9,257,620	22.53	167.0	123.5	1.24
9 Sep	714	700	-14	10,228,738	6.98	160.7	119.1	1.25

SUPPLEMENTARY FIGURE S1: Categories of persons present in the FluxVision database.

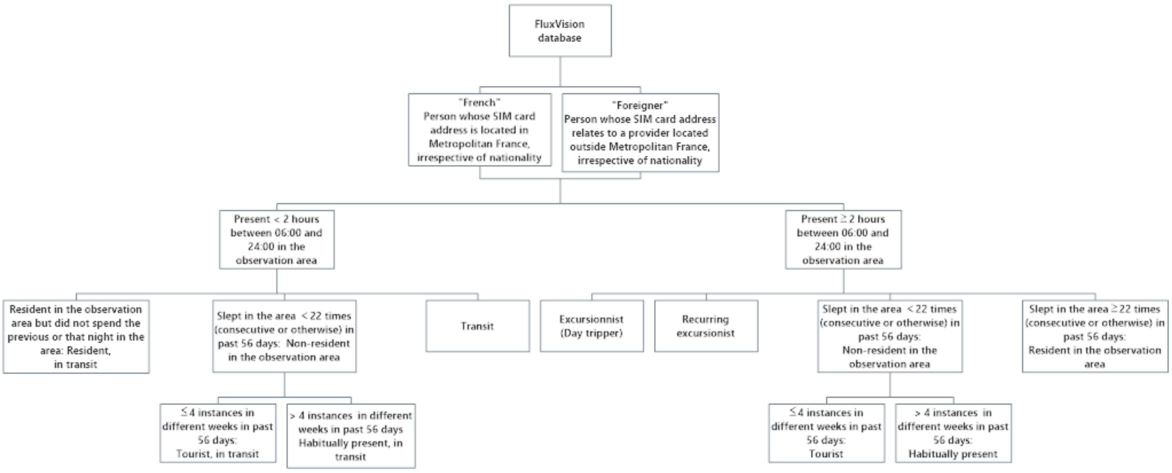

Supplementary Figure S2: SpF and corrected SARS-CoV-2 incidence by moving week using positive tests done by IdF residents and habitual visitors in IdF as numerator and the number of IdF residents estimated using cell phone data as the denominator, Île-de-France, 28 June–9 September 2021

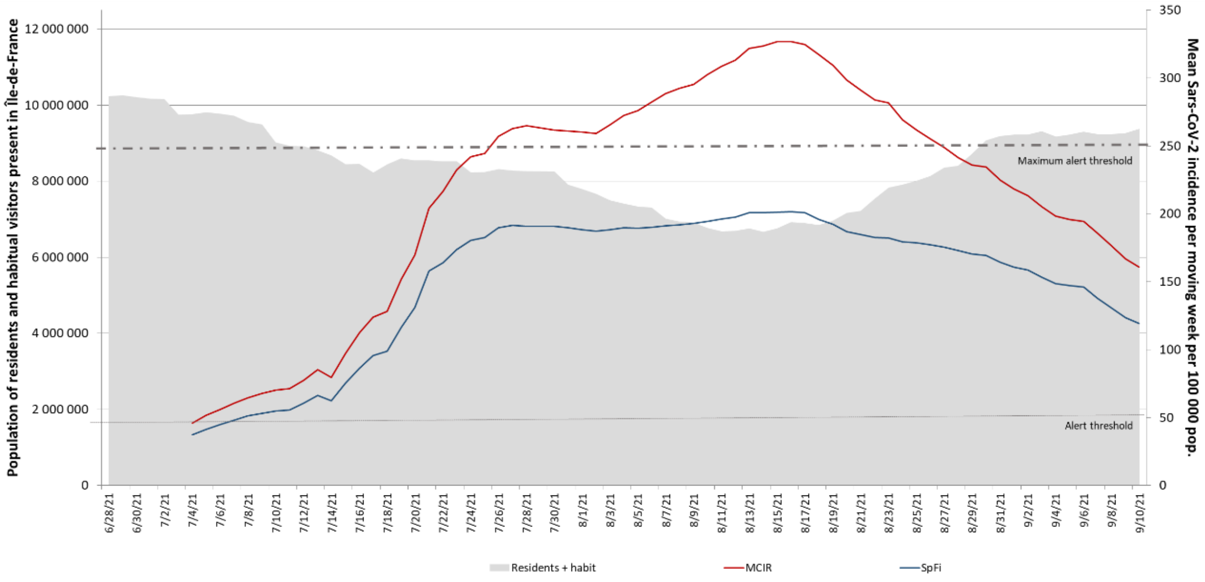

Data from 28 June–9 September were used for computations.
